# Status of Healthcare in LGBTQI+ Community in Nepal: Challenges and Possibilities

**DOI:** 10.31729/jnma.7948

**Published:** 2023-01-31

**Authors:** Pratik Subedi, Anjali Jha

**Affiliations:** 1 Nepalese Army Institute of Health Sciences, Sanobharyang, Kathmandu, Nepal

**Keywords:** *healthcare*, *LGBTQ persons*, *sexual minorities*

## Abstract

Lesbian, gay, bisexual, transgender, queer, and intersex are at increased risk of getting infected with sexually transmitted infections, suicidal behaviours, and substance and physical abuse. Stigmatization and discriminatory attitudes toward the community have led to disparities while receiving healthcare. In this article, we discuss the condition of healthcare in sexual minorities in Nepal, the major barriers to accessing healthcare facilities, the roles played by nongovernmental organizations, and the possible ways to improve healthcare in the lesbian, gay, bisexual, transgender, queer, and intersex community.

## INTRODUCTION

Lesbian, gay, bisexual, transgender, queer, and intersex (LGBTQI+) is an initialism referring to lesbian (homosexual woman), gay (homosexual man or woman, used primarily for a man), bisexual (person attracted to both genders), transgender (a person whose gender identity doesn't align with the sex they are assigned at birth), queer (synonymous to gay, used to empower themselves) and intersex (person with both male and female biological traits).^1^ Research has shown that LGBTQI+ are at increased risk of sexually transmitted infections (STIs), suicidal behaviours, and physical and substance abuse.^2^ Moreover, discriminatory attitudes and practices in healthcare centres restrain them from seeking healthcare.^2^ In this article, we discuss the status of healthcare for LGBTQI+ in Nepal, its challenges and possibilities.

## LGBTQI+ HEALTH PROBLEMS

LGBTQI+ youths are affected by gonorrhoea, chlamydia, HIV, and other reproductive health problems at increased rates than heterosexuals owing to an increased number of sexual partners, early onset of sexual intercourse, and unprotected anal sexual intercourse.^3^ Moreover, exclusion from the workplace due to existing cultural norms and poverty experienced by trans people lead them to unsafe practices like prostitution which makes them more susceptible to STDs.^2^ Social stigmatization, peer victimization, family rejection and verbal and physical harassment have led to a greater incidence of depression, anxiety, suicidal behaviours, and substance abuse in sexual minority adolescents than in heterosexual adolescents.^4^

## STATUS OF HEALTHCARE IN LGBTQI+ COMMUNITY IN NEPAL

Inherently, LGBTQI+ are already at greater reproductive, physical, and mental health risks, and the healthcare services offered to them are even poorer than heterosexuals. Healthcare providers are not far from the cultural norms of society, thus exhibiting discriminatory attitudes and stigmatization of sexual minorities which is one of the major barriers to acquiring healthcare services.

The medical curriculum in Nepal is flawed as well, research has shown that the knowledge and attitudes of Nepalese medical students toward LGBTQI+ are very poor.^5^ The health professionals of tomorrow will not be able to provide better healthcare to sexual minorities if they see them with disgust. In Nepal, medicine is taught in either colleges under Tribhuvan University and Kathmandu University or BP Koirala Institute of Health Sciences or Patan Academy of Health Sciences, or the recently formed Karnali Academy of Health Sciences, all of which has a different curriculum. Though there are five different curriculums or five different ways MBBS is taught in Nepal, all of them see heterosexual as a norm. The patient history form has options of either male or female only, so LGBTQI+ community are reluctant to open up to the healthcare provider which further complicates clinical diagnosis.

Nepal has been touted as the beacon of LGBTQI+ rights in South Asia ever since the supreme court's verdict in 2007 guaranteed legal recognition of the third gender and antidiscriminatory laws against LGBTQI+.^6^ Though Nepal's policies look good on paper, implementation has not been better. People find it difficult to change their citizenship certificate after their trans-surgery even in Kathmandu, thus inviting legal issues throughout their lifetime. Officials do not allow people to go abroad to take part in health conferences just because their passport photo does not match their appearance.^7^ Even in the hospitals, the wards are gender assigned to either male wards or female wards, thus administrative staff in the hospital can't figure out where to admit the transgender. The patients also feel uncomfortable visiting hospitals as they believe their existence is denied, this leads to selfexclusion.

However, different nongovernmental organizations (NGOs), international nongovernmental organizations (INGOs), and social organizations have done a good job in imparting health education to sexual minorities without government support. Studies have shown the utilization of condoms and knowledge and attitudes of sexual minorities regarding HIV/AIDs to be pretty good.^8,9^ However, these studies were conducted only in Kathmandu valley, a privileged area as compared to other districts. This was the basic limitation of these studies. One of these organisations, Blue Diamond Society (BDS) has been educating Nepalese society regarding sexual health, advocating for the rights of sexual minorities, documenting violence against LGBTQI+ youth and providing health services to them. Most of the people in LGBTQI+ community have experienced a disparity in hospice and palliative care.^10^ So, one of their health programs is an LGBTQI+ hospice and palliative care facility renowned as "BDS care and support centre". The centre provides support to LGBTQI+ community members of Nepal having chronic or terminal illnesses free of cost. The healthcare professionals at "BDS care and support centre" have found that the needs of sexual minorities in palliative and hospice care are multifaceted, they not only require palliation of physical, emotional, and spiritual pain but also their social and structural causes of pain.^11^ Most of the patients receiving care there reported being stigmatized by family members as soon as they learned about their disease leading to self-exclusion. The fear of dying without family adds to the already present trauma. A healthcare professional has to not only follow the duty of a clinician but also of a family member in times of being excluded by family members.

## POSSIBILITIES OF LGBTQI+ HEALTHCARE IN NEPAL

**Improving the cultural understanding of LGBTQI+ in Nepal**

Nepal has a supportive legal system and religious point of view, still, it is culturally conservative in terms of sexuality. Social traditions see only two genders, male and female. Other expressions of sexuality are deemed to be a bad influence of westernization. People fail to understand the deep psychological difficulties the LGBTQI+ community face and some even treat them as insects. Having an open dialect with people in the community, making them understand the sufferings faced by sexual minorities, and making them realize that all they ask for is love and care is imperative at the moment to reduce stigmatization and self-exclusion.

**Training health professionals to be sensitive about gender issues**

The medical curriculum in Nepal is outdated and recognizes heterosexuals as the only possible patients. The curriculum should include classes that would improve the knowledge, attitudes, and practices of medical students. They should be trained in a way they are gender sensitive to their patients and should acknowledge the LGBTQI+ community during history taking as well.

**Establishment of healthcare units, especially for LGBTQI+ people**

Palliative care is interdisciplinary ranging from medicines for physical symptoms to chaplains for spiritual and emotional support. Patients find peace when they see people of their own community around them. The BDS care and support centre located in Lazimpat has been providing exemplary services to the sexual minority, but the country requires more such units to be established in every province at least.

**Appointment of LGBTQI+ health professionals to serve these centres**

Holistic care includes both medical and non-medical components. Meeting healthcare professionals who come from their own backgrounds and hearing their stories would make patients comfortable. The fear of stigmatization and discrimination would also go away improving the overall experience.

**New identification card for transgender people after their surgery**

It is important to give new identification cards viz. citizenship certificates to trans people after their surgery. Just because their appearance doesn't match their photo and gender on the identification card, they are unable to benefit from the available services.

**Awareness for the entire community about sexual health**

Evidence has shown awareness and educational programs have improved health practices in the LGBTQI+ community. In order to utilize the services offered to them, they need to understand their own sexual health and the risks associated with it. The awareness can be done through open lectures, seminars, workshops, etc. Until now, most of the awareness is given by NGOs and INGOs only in Kathmandu and other privileged areas. Awareness campaigns should also be done in rural areas, where people lack knowledge and resources.

## WAY FORWARD

Healthcare in the LGBTQI+ community is a topic overlooked for years in Nepal. There exists a wide research gap, as the research conducted so far is mostly Kathmandu centric giving us insufficient information about the real condition of sexual minorities in the country. More research has to be conducted for assessing the real situation and making plans accordingly. Also, in a country that claims to be the most progressive for LGBTQI+ rights in South Asia, it becomes imperative for the people acting at the governance level, law formulations, medical professionals, and every individual, to change their attitudes and work together to ascertain equal healthcare services to the minorities eliminating all the disparities they have been facing.
